# Cognitive process underlying ultimatum game: An eye-tracking study from a dual-system perspective

**DOI:** 10.3389/fpsyg.2022.937366

**Published:** 2022-09-27

**Authors:** Zi-Han Wei, Qiu-Yue Li, Ci-Juan Liang, Hong-Zhi Liu

**Affiliations:** ^1^Key Research Base of Humanities and Social Sciences of the Ministry of Education, Academy of Psychology and Behavior, Tianjin Normal University, Tianjin, China; ^2^Faculty of Psychology, Tianjin Normal University, Tianjin, China; ^3^Department of Social Psychology, Zhou Enlai School of Government, Nankai University, Tianjin, China; ^4^Laboratory of Behavioral Economics and Policy Simulation, Nankai University, Tianjin, China

**Keywords:** ultimatum game, dual-system, eye-tracking, cognitive effort, attention allocation

## Abstract

According to the dual-system theories, the decisions in an ultimatum game (UG) are governed by the automatic System 1 and the controlled System 2. The former drives the preference for fairness, whereas the latter drives the self-interest motive. However, the association between the contributions of the two systems in UG and the cognitive process needs more direct evidence. In the present study, we used the process dissociation procedure to estimate the contributions of the two systems and recorded participants eye movements to examine the cognitive processes underlying UG decisions. Results showed that the estimated contributions of the two systems are uncorrelated and that they demonstrate a dissociated pattern of associations with third variables, such as reaction time (RT) and mean fixation duration (MFD). Furthermore, the relative time advantage (RTA) and the transitions between the two payoffs can predict the final UG decisions. Our findings provide evidence for the independent contributions of preference for fairness (System 1) and self-interest maximizing (System 2) inclinations to UG and shed light on the underlying processes.

## 1. Introduction

Fairness-related decision-making has drawn much attention in the past decades (Fehr and Schmidt, 1999; Camerer, 2003). Ultimatum game (UG) is widely used in the literature to investigate the underlying mechanisms of human fairness (Gth et al., 1982; Fabre et al., 2016; Gong et al., 2017; Vavra et al., 2018; Matarazzo et al., 2020). In a typical UG, two players share a sum of money. One player is assigned the role of a proposer and is given a sum of money to split between them two. The other player is assigned the role of a responder who has to decide whether to accept or reject the proposed offer. If the responder agrees with the proposal, then the money will be divided in accordance with the offer. If the responder rejects the proposal, then neither player receives anything (Gth et al., 1982).

The classical economic model suggests that individuals are rational and driven by self-interest (Friedman and Savage, 1948; Kahneman et al., 1986), which means that the proposer would offer the smallest amount and that the responder would accept any non-zero offer. However, numerous studies have demonstrated that both players are likely to systematically disregard these rational predictions and behave fairly. Commonly, most proposers offer 40–50% of the total amount, whereas responders tend to reject offers lower than 30% (Gth et al., 1982; Gth and Tietz, 1990; Camerer and Thaler, 1995; Nowak et al., 2000). Thus, the UG marks an anomaly because it challenges these traditional theories about human behavior (Thaler, 1988).

### 1.1. Dual-system in UG

Recent studies have attempted to understand the mechanism underlying UG decisions from the perspective of dual-system theories (Sanfey and Chang, 2008; Halali et al., 2014; Hochman et al., 2015), which have received much theoretical consideration in the field of judgment and decision-making (Evans, 2003; Lieberman, 2007). Dual-system theories are assumed to characterize choice behavior and human cognition as governed by the interaction between two independent systems (Sloman, 1996; Stanovich and West, 2000; Kahneman and Frederick, 2002). The first is System 1, which is assumed to be automatic, fast, and effortless, and the second is System 2, which is assumed to be slower and more controlled, effortful, and deliberative. According to the evolutionary game-theoretic model (Bear and Rand, 2016), System 1 is built on the beliefs derived from a preference for fairness, whereas System 2 focuses on self-interest maximizing. Moreover, the rejection of an unfair proposal in UG is an automatic response controlled by System 1, whereas the acceptance of an unfair proposal is driven by the self-interest motive in System 2 (Bear and Rand, 2016).

Several studies have reported that manipulating the factors that can affect the process of System 1 could change individuals' choices in UG. For instance, time constraints (Cappelletti et al., 2011; Grimm and Mengel, 2011; Neo et al., 2013) or the depletion of cognitive control resources (Halali et al., 2014) increased the rate of rejected unfair offers in UG. These findings can be interpreted as individuals being more likely to adopt the automatic System 1 when their cognitive resources are limited, thereby proving the existence of that system. However, to our knowledge, the existence of System 2 and the independent contributions of these two systems still lack direct evidence.

### 1.2. Process dissociation procedure

Process dissociation procedure (PDP) is a general approach to estimate the contributions of an automatic process (System 1) and a controlled process (System 2) (Jacoby, 1991; Ferreira et al., 2006, 2016). Process dissociation procedure has been used successfully in judgment and decision-making under uncertainty (Ferreira et al., 2006, 2016; Damian and Sherman, 2013; Mata, 2016), moral decisions (Conway and Gawronski, 2013; Mata, 2019), and purchase decisions (Jami and Mishra, 2014). The fundamental logic of PDP is to design experiments that include two conditions: the inclusion condition, in which System 1 and System 2 converge on the same conclusion, and the exclusion condition, in which the conclusion of one system is different from the conclusion of the other system. By assuming that both systems contribute to performance and operate independently, the contributions of each system can be estimated by comparing performance across the two conditions (Ferreira et al., 2006).

To illustrate this idea, consider a UG situation wherein the proposer offers an unfair proposal (e.g., the proposer gets $8 and the responder gets $2). In this situation, System 1, which shows disadvantage unfairness aversion, will predict rejection, whereas System 2, which shows self-interest, will predict acceptance. Consequently, Systems 1 and 2 reach different conclusions, and we have an exclusion condition. However, when the proposal is that the proposer and responder receive $4 and $6, respectively, Systems 1 and 2 predict an acceptance response and concur on the same conclusion. In this situation, we have an inclusion condition.

By having the inclusion and exclusion conditions, we can estimate the independent contributions of Systems 1 and 2. If the contributions of Systems 1 and 2 to a decision task is defined as (A) and (C), respectively, then the probability of going with the recommendation of System 1 but not with the recommendation of System 2 in an exclusion condition is equal to the probability of using System 1 given that System 2 is not used:


(1) $$\begin{array}{l} P\left(exclusion\right)=P_{A}-\left(P_{A}\times P_{C}\right). \end{array}$$

When both systems agree with each other in the inclusion condition, the probability of taking action on the dominant option recommended by both systems equals the probability of using System 1 plus that of using System 2 minus the probability of using both systems:


(2) $$\begin{array}{l} P\left(inclusion\right)=P_{A}+P_{C}-\left(P_{A}\times P_{C}\right). \end{array}$$

Accordingly, we can estimate the independent contributions of System 1 (*P*_*A*_) and System 2 (*P*_*C*_) using the following equations (Ferreira et al., 2006):


(3) $$\begin{array}{l} P_{C}=P\left(inclusion\right)-P\left(exclusion\right), \end{array}$$

(4) $$\begin{array}{l} P_{A}=P\left(exclusion\right)/\left(1-P_{C}\right). \end{array}$$

### 1.3. Eye-tracking technique in UG

In recent years, research on human decision-making has expanded from purely behaviorist approaches that focus on decision outcomes to include more cognitive approaches focusing on the decision process that occurs prior to the response (Glaholt and Reingold, 2011). Among the process-tracing methodologies, the eye-tracking technique has been successful in studying the complex cognitive activities involved in decision-making (Sui et al., 2020; Liu et al., 2021a,b; Zhou et al., 2021). This technology allows decision-makers to freely investigate information while providing researchers a way to measure the information uptake process (Just and Carpenter, 1984; Ashby et al., 2016b). The eye-tracking technology has also been proven to be a powerful tool for capturing cognitive processes involved in decision-making (Glaholt and Reingold, 2011; Ashby et al., 2016b).

In the past years, researchers have attempted to examine the characteristics of eye movement during a UG. For instance, Colombo et al. (2013) investigated participants' eye movements while playing a UG. In the experiment, participants played the role of proposers, and they were matched with responders who could either be sincere or lying. Their results revealed that participants' eye-tracking measures (e.g., first fixation duration on the partner and total fixation count) differed between the sincere and lying conditions. Villani et al. (2013) examined the eye movements of responders in a UG, which is more relevant to the current research. They found that responders looked at their own outcome and that of the proposer at a much longer time when the proposal was fair than when it was unfair.

However, two aspects can be further examined by using the eye-tracking technique. First, the association between the contributions of the two systems in UG and the cognitive process should be tested. In this case, the contributions of the two systems in UG can be quantitatively estimated using the PDP paradigm. Moreover, the eye-tracking technique allows the measures to reflect the cognitive process. This case enables us to examine the relationship between the contributions of the two systems and the cognitive processing measures.

Second, the association between the eye-tracking measures and the decision-making in UG can also be explored. The eye-tracking measures can reflect rich information about the cognitive process during decision-making and are sensitive to the final choices (Liu et al., 2020, 2021b; Zhou et al., 2021). To our knowledge, few studies have tested the predicting effect of eye-tracking measures on UG decisions. Examining this predicting effect can help explain the underlying mechanism of UG at the trial level.

### 1.4. Hypotheses

Accordingly, the present research constructed the inclusion and exclusion conditions to estimate the contributions of the two systems in UG and recorded participants' eye movements, focusing on the questions raised above. Our hypotheses are thus derived.

First, we assumed that the contributions of the two systems correlate with the cognitive process. On the one hand, previous studies have shown that individuals under time constraints exhibited more decisions predicted by intuitive System 1 (Sutter et al., 2003; Cappelletti et al., 2011), indicating that the reaction time (RT) is related to the contribution of System 1. On the other hand, the deliberative System 2 is usually accompanied by high cognitive effort during decision-making (Horstmann et al., 2009; Su et al., 2013; Zhou et al., 2021). The mean fixation duration (MFD), which is the average duration of single fixations during a decision, can reflect the cognitive effort level (Velichkovsky, 1999; Horstmann et al., 2009; Amblee et al., 2017). Thus, it is assumed that the MFD is related to the contribution of System 2. Therefore, we hypothesized that the RT correlates negatively with the contribution of System 1, whereas the MFD correlates positively with the contribution of System 2 (H_1_).

Second, we also assumed that the eye-tracking measures could predict the UG decisions at the trial level. The drift-diffusion model (DDM) assumes that preferences are constructed through a stochastic dynamic information acquisition process (Ratcliff, 1978). Specifically, an individual's preference is formed by sampling from options until the evidence supporting one option is strong enough to induce judgment (Raab and Johnson, 2007; Ashby et al., 2016a). According to the DDM, individuals are more likely to choose the option that they looked for a longer time (Krajbich et al., 2010; Sui et al., 2020; Liu et al., 2021a). Following the same logic, we assumed that the relative time advantage (RTA) could predict the UG decisions (H_2_). Specifically, the longer the responders looked at their own payoffs, the more likely they accept the proposals. In addition, the transitions between the two payoffs may predict the UG decisions. In this case, the transitions between the two payoffs indicated the sensitivity to the fairness of the proposal. The more the responder cares about the fairness of a proposal, the more likely he/she will reject the proposal. Thus, we assumed that the transitions between the two payoffs positively predict the reject response (H_3_).

## 2. Methods

### 2.1. Participants

We used G*Power (Faul et al., 2007) to calculate the sample size needed to achieve 80% power to detect the correlation effect of *r* = 0.25 (small effect), using a correlation test at the 0.05 significance level. The necessary sample size was *N* = 97. Therefore, 101 participants (*M*_*age*_ = 21.5 2.2; 57% female) were recruited from a university's human subjects pool and were asked to take part in the study. They were given 5 Yuan (RMB; approximately US$0.8) in cash for their participation. All the participants had a normal or corrected-to-normal vision, and they had provided prior written informed consent.

### 2.2. Apparatus

The stimuli were presented on a 17-inch LCD monitor with a display resolution of 1,024 × 768 pixels and a refresh rate of 60 Hz. The participants responded during the experiment by pressing on specific keys on the keyboard. The distance between the screen and the participants' eyes was 60 cm. Viewed from this distance, the screen covered a visual angle of 36° horizontally and 29° vertically. The participants' eye movements were recorded by an EyeLink 1000 plus (SR Research) eye tracker with a sample rate of 1,000 Hz. A chin rest was used to reduce head movement. As both eyes fixate on the same spot, it is sufficient to record the movements of one eye. Experimental data were collected and processed by the Experiment Builder (version 2.3.38) and Data Viewer software (version 4.2.1).

### 2.3. Experimental task and procedure

First, the participants must consent to participate in the experiment. Thereafter, they were given instructions explaining the rules of the UG and a brief description of the apparatus. They were told that they would play as a responder in the UG and would receive offers from other participants in previous experiments in the lab. Then, the participants were asked to accept or reject each offer. To improve the plausibility of the story, they completed a questionnaire in which they provided offers for 20 anonymous partners. They were asked to decide how to divide 30 yuan between themselves and their partner. To motivate the participants' choices, they were told that at the end of the experiment, one of the trials in which they accepted the offer would be selected randomly by the computer and that they would be paid based on that trial. This experimental design is commonly used in UG research (Gaertig et al., 2012; Wei et al., 2018; Pei et al., 2021). We also verbally asked the participants whether they had any questions about the content of the experiment and no one reported that they had doubts about the cover story.

The stimuli comprised 20 pairs of payoffs, including 10 inclusive payoffs and 10 exclusive payoffs. Details can be found in Table S1 in Supplementary materials (https://osf.io/xz7ms). The initial amount of the proposer was always 30 yuan. In the inclusive payoffs, the responder's payoff was no less than the proposer's payoff; thus, Systems 1 and 2 predicted the “accept” decision. By contrast, in the exclusive payoffs, the responder's payoff was less than the proposer's payoff; thus, System 1 predicted the “reject” decision, but System 2 predicted an “accept” response. The two payoffs (i.e., the responder and proposer's payoffs) were shown on the screen, and the placement of the payoffs was counterbalanced across participants. Specifically, half the participants saw the responder's payoff as the top number, and the other half saw the proposer's payoff as the top number. The center-to-center distance between the two pieces of information is greater than 5°, which ensures the proper fixation of the information. Moreover, the peripheral identification of adjacent information is not possible (Rayner, 1998, 2009). The options were presented in randomized order for each participant.

**Figure 1:**
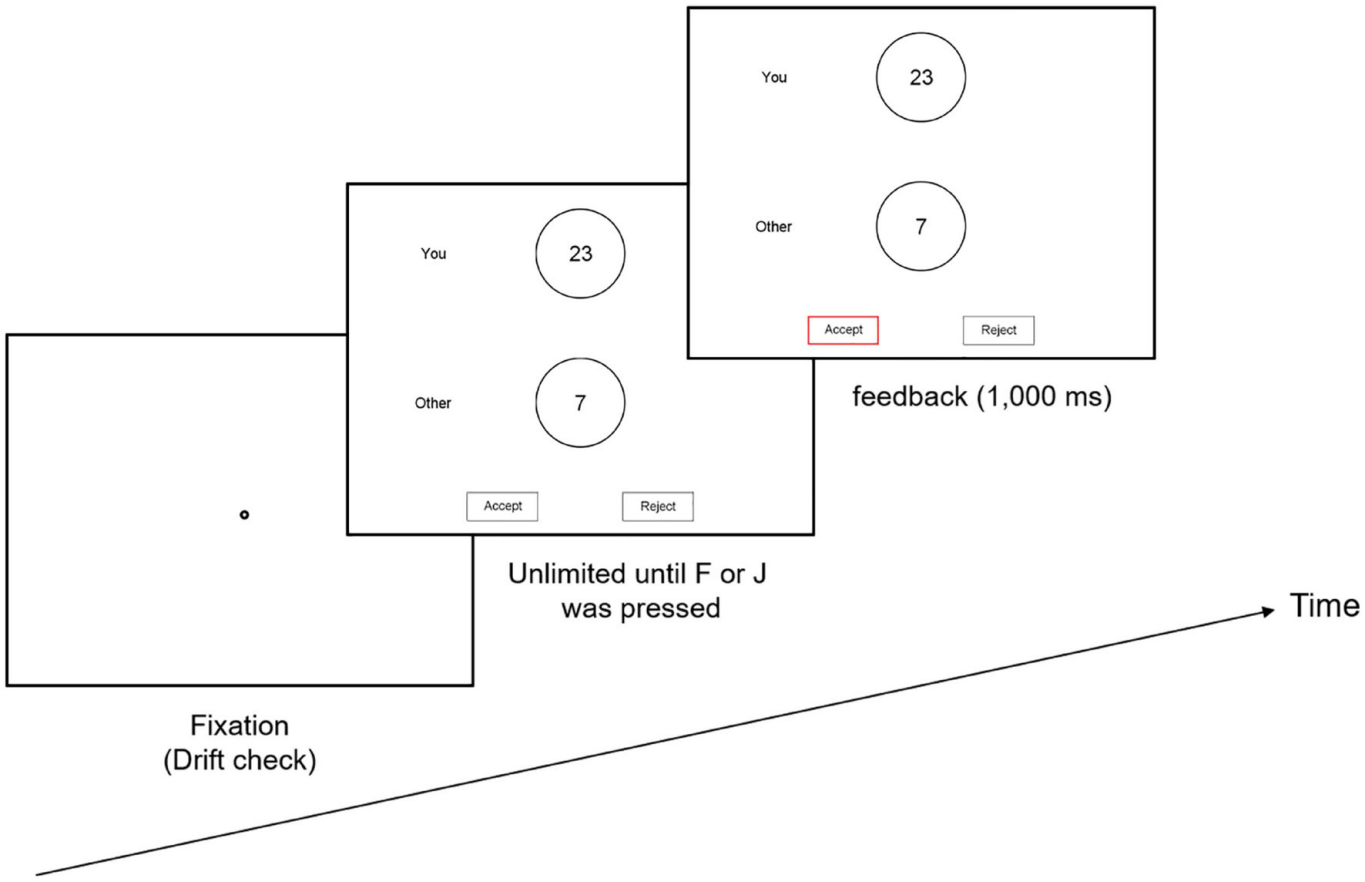
Trial procedure and timing in the experiment. Each trial began with a fixation in the middle of the screen. After each response, a 1,000-ms intertrial interval with a feedback screen was presented before the next trial began.

Each participant was calibrated to the eye tracker using the five-dot calibration method at the start of the experiment and was recalibrated as needed (e.g., if the drift check failed). The maximum error for validation was 0.5 degrees of visual angle. After the initial calibration, two practice trials were performed to familiarize the participants with the task. At the beginning of each trial, a fixation disc appeared at the center of the display. The disc was also used as a drift check for the eye tracker. When a fixation on the disc was registered, the participants pressed the space bar to begin the presentation of the payoffs. They were instructed to press “F” to accept the proposal or to press “J” to reject the proposal. There was no time limit, and the screen was cleared once a participant pressed a button. After the participant responded, a feedback screen was presented for 1,000 ms. Figure 1 presents the details.

### 2.4. PDP parameters

For each participant, we first calculated the respective probabilities of acceptance of respondents in the inclusion and the exclusion conditions. Then, we calculated the PDP scores of *P*_*A*_ and *P*_*C*_ using the two algebraic formulas presented above. If someone never chose a System 1 answer in the exclusion condition and always accepted the offers in the inclusion condition, the calculation of the individual's estimate of *P*_*A*_ was mathematically constrained. Thus, two participants were removed from the analysis.

### 2.5. Eye-tracking measures

#### 2.5.1. Preprocessing of eye-tracking data

The collected eye movement data were analyzed by using EyeLink Data Viewer (SR Research, Ontario, Canada). In the task, two non-overlapping, identically sized rectangular regions of interest around each piece of information were defined. Fixations were defined as periods of a relatively stable gaze between two saccades. However, fixations shorter than 50 ms were excluded from the analyses.

#### 2.5.2. Eye-tracking measures

The following three eye-tracking measures were used to test our hypotheses. The first measure is the MFD, which is calculated by adding the duration of all fixations during a trial and dividing the total by the number of fixations. The value of MFD is sensitive to cognitive effort (Amblee et al., 2017). The second measure is the RTA, which is an index of attention allocation (Krajbich et al., 2010; Prnamets et al., 2015). In the current work, the RTA was computed as follows:


(5) $$\begin{array}{l} \begin{array}{l} RTA=\frac{gaze\;time\;on\;the\;responder^{\prime }s\;payoff-gaze\;time\;on\;the\;proposer^{\prime }s\;payoff}{gaze\;\;time\;on\;the\;responder^{\prime }s\;payoff+gaze\;time\;on\;the\;proposer^{\prime }s\;payoff} \end{array} \end{array}$$

The higher value of RTA indicates that the responder's payoff received more attention than the proposer's payoff. The final measure is the gaze-shift frequency (GSF), which indexes how frequently the gaze shifted back and forth among the payoffs presented on the screen (Folke et al., 2016), indicating sensitivity to the fairness of the proposal.

## 3. Results

Data from the experiment reported in this article are publicly available via the Open Science Framework (https://osf.io/xz7ms).

### 3.1. UG characteristics

Overall, the proposals were accepted on 93% (*SD* = 17) of the inclusion condition and 50% (*SD* = 25) of the exclusion condition. The difference between the two conditions was statistically significant (*t*_98_ = 12.11, *p* < 0.001, Cohen's *d* = 1.98). Participants also took a longer time to respond to the exclusion condition (*M* = 1.99 s, *SD* = 0.72) than to the inclusion condition (*M* = 1.45 s, *SD* = 0.80) (*t*_98_ = 6.40, *p* < 0.001, Cohen's *d* = 0.70).

### 3.2. PDP analysis

**Figure 2:**
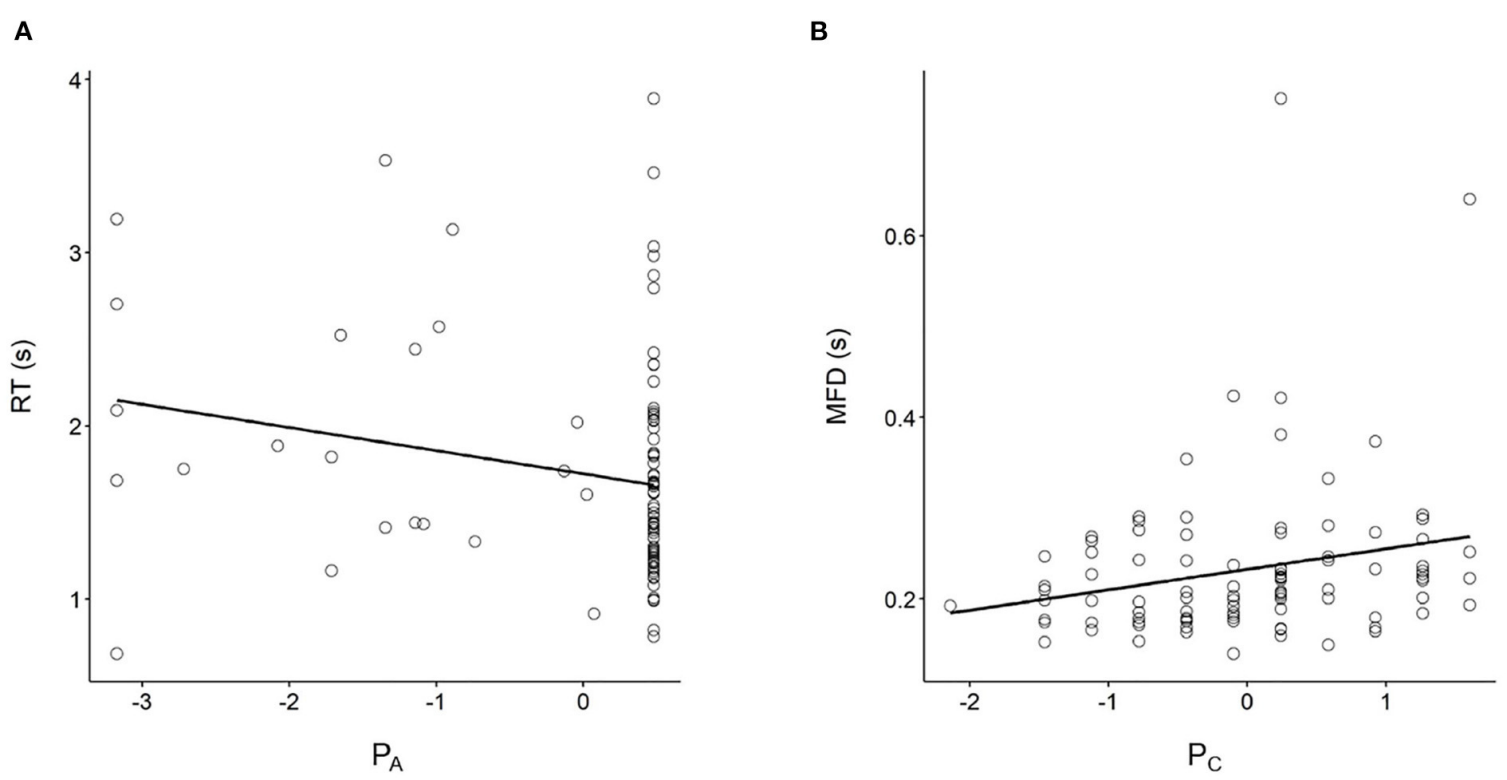
Scatter plots of the PDP parameters and behavioral data. **(A)** RT correlated negatively with the *P*_*A*_ parameter; **(B)** MFD correlated positively with the *P*_*C*_ parameter.

Table 1 presents the results of the correlation analyses. As can be seen, the two PDP parameters were not correlated (*r* = 0.01, *p* = 0.888). This is consistent with the assumption that Systems 1 and 2 are independent rather than inversely related. The correlation analysis also revealed that RT correlated negatively with the *P*_*A*_ parameter (*r* = –0.21, *p* = 0.037, see Figure 2A), but not with the *P*_*C*_ parameter (*r* = 0.13, *p* = 0.201). This result indicated that more contribution of System 1 can be reflected by shorter RTs. This is consistent with H_1_. The results also showed that the MFD correlated positively with the *P*_*C*_ parameter (*r* = 0.22, *p* = 0.026, see Figure 2B) but not with the *P*_*A*_ parameter (*r* = 0.11, *p* = 0.288). This result indicated that more contribution of System 2 can be reflected by longer fixation duration. This is also consistent with our H_1_.

**Table 1:** Correlation coefficient matrix of PDP scores and cognitive measures.

**Variable**	***P*_*A*_**	***P*_*C*_**	**RT**	**MFD**
*P*_*A*_	1		
*P*_*C*_	0.01	1	
RT	−0.21^*^	0.13	1
MFD	0.11	0.22^*^	0.03	1

^*^p < 0.05.

### 3.3. Predictive effect of the eye-tracking measures

**Figure 3:**
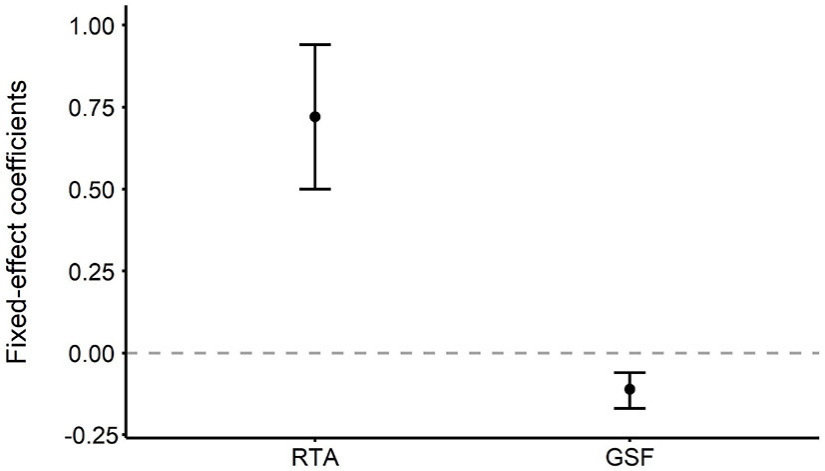
Unstandardized regression coefficients for the effects of RTA and GSF on choice behavior. Both the RTA and the GSF were significant factors in predicting UG decisions. Error bars indicate 95% confidence intervals.

To examine the predictive effect of the eye-tracking measures on UG decisions, we applied the mixed effect logistic models with the random effects of the participant and trial number to analyze our data, using the lme4 and lmerTest packages in the R statistical environment (Bates et al., 2015; Kuznetsova et al., 2017). The UG decision (1 = accept, 0 = reject) was a dummy-coded dependent variable. The values of RTA and GSF were entered as fixed effects. The results indicated that the RTA was a significant factor in predicting the choices (*b* = 0.74, 95% CI = [0.53, 0.96], *z* = 6.73, *p* < 0.001), thus implying that participants were more likely to accept the proposals when they looked at their own payoffs longer. Furthermore, the GSF was significant in predicting the choices (*b* = –0.11, 95% CI = [–0.17, –0.06], *z* = –3.82, *p* < 0.001), indicating that the likelihood of rejecting the proposals is great when the transitions between the two payoffs are substantial. Therefore, H_2_ and H_3_ are supported. Figure 3 shows the details.

## 4. Discussion

In this study, we used eye-tracking technology to examine the mechanisms underlying decisions in a UG. By using the PDP paradigm, we estimated the contributions of automatic System 1 and controlled System 2. At the individual level, we found that the contribution of System 1 correlated negatively with RT, while that of System 2 correlated positively with MFD. We further tested the predictive effect of eye-tracking measures at the trial level. The results revealed that (1) the more attention allocated to the participants' (responders') payoffs, the more likely that the offer would be accepted; and (2) the more transitions between the two payoffs, the more likely that the offer would be rejected.

Our findings provide evidence for the dual system in UG. To our knowledge, this analysis is the first to use the PDP paradigm in UG research. By using this paradigm, we delineated the independent contributions of automatic System 1, which drives the preference for fairness, and those of controlled System 2, which drives the self-interest motive. The two parameters were uncorrelated, demonstrating a dissociated pattern of correlations with third variables, such as RT and MFD. These findings imply that the two independent systems exist in the choice of UG. In addition, the estimated contributions can be reflected in the cognitive process, thereby supporting the evolutionary game-theoretic model (Bear and Rand, 2016). Future research may apply the PDP paradigm to explore the dual system in other games, such as dictator game and the sender-receiver game (for a review, see Capraro and Perc, 2021).

Furthermore, our findings are consistent with previous evidence from neuroscience research. For instance, event-related potential (ERP) research on UG revealed that unfair offers provoked more brain activities in feed-back-related negativity (FRN) (Boksem and Cremer, 2010; Hewig et al., 2011). This can be interpreted to reflect activities related to the anterior cingulate cortex (ACC), a region considered a part of System 2, which is thought to apply cognitive control to resolve conflicts (Boksem and Cremer, 2010). A functional magnetic resonance imaging (fMRI) study indicated that the affective and deliberative processes activated distinct neural areas (Sanfey et al., 2003). The authors also found that activation in the anterior insula, which is the effective part of the brain, exhibited a positive correlation with the rejection proportion of unfair offers in UG. By contrast, the acceptance proportion of the unfair offers in UG has been attributed to the right dorsolateral prefrontal cortex, which is the cognitive part of the brain. Our findings suggest the feasibility of testing the correlation between the contributions of the two systems and the cognitive process. Future research might further examine the relationship between the two parameters estimated by the PDP paradigm and the neural activities.

Another implication of our findings is that the decision of UG might be constructed through a dynamic information acquisition process. The DDM assumes that individuals' decisions are formed by sampling from available options until the evidence supporting one option is strong enough to induce a judgment (Ratcliff and Smith, 2004; Ashby et al., 2016a; Smith and Krajbich, 2018). This class of process models has shown great promise in terms of the ability to predict risky choices (Busemeyer and Townsend, 1993), intertemporal choice (Dai and Busemeyer, 2014), and decisions on consumer products (Krajbich et al., 2010; Krajbich and Rangel, 2011). The predictive effect of RTA revealed in the present study indicates that the decisions in UG might also be an evidence accumulation process, which is motivated by the fixations. Future research may further explore the applicability of DDM in UG decisions and attempt to model the UG decisions by using fixations.

In addition, the findings that the transitions between the payoffs predict the UG decisions also have implications for the cognitive process in UG. Previous research have shown that the transitions between payoffs can reflect the fairness perception (Jiang et al., 2016) and correlate with social preference (Fiedler et al., 2013). Consistent with the literature, we found that the transitions between payoffs are also correlated with the fairness sensitivity in UG and can predict the UG decisions. These findings suggest that the information of individuals' own payoffs and the proposers' payoffs in a UG may not be processed independently and that the fairness of the offer might be evaluated in its entirety.

It should be noted that there might be other motivations during UG decisions. In the inclusion condition, the responders receive more money than the proposers, and there should be no disadvantage unfairness aversion. In this situation, the responders should accept all the payoffs. However, our results showed that almost 7% of all the payoffs in the inclusion condition were rejected. Therefore, this finding suggests that there might be other motivations for rejection decisions, such as advantageous inequity aversion (e.g., Yu et al., 2021), which should be further examined in future research.

In conclusion, the present study provided evidence for the independent contributions of preference for fairness and self-interest maximizing inclinations to UG, thus shedding light on the underlying processes.

## Data availability statement

The original contributions presented in the study are included in the article/Supplementary material, further inquiries can be directed to the corresponding author/s.

## Ethics statement

The studies involving human participants were reviewed and approved by Institutional Review Board of Psychology of Nankai University. The patients/participants provided their written informed consent to participate in this study.

## Author contributions

Z-HW and H-ZL conceived and designed this study and wrote the paper. Z-HW, Q-YL, and C-JL designed experimental stimuli and procedures. C-JL implemented experimental protocols and collected data. Q-YL and H-ZL analyzed data. All authors contributed to the article and approved the submitted version.

## Funding

This work was partially supported by the National Natural Science Foundation of China (Nos. 72001158 and 71901126), the Humanity and Social Science Youth Foundation of Ministry of Education of China (No. 19YJC190013), and the Fundamental Research Funds for the Central Universities (No. 63222045).

## Conflict of interest

The authors declare that the research was conducted in the absence of any commercial or financial relationships that could be construed as a potential conflict of interest.

## Publisher's note

All claims expressed in this article are solely those of the authors and do not necessarily represent those of their affiliated organizations, or those of the publisher, the editors and the reviewers. Any product that may be evaluated in this article, or claim that may be made by its manufacturer, is not guaranteed or endorsed by the publisher.
