# Supplementary material for: Cognitive process underlying ultimatum game: An eye-tracking study from a dual-system perspective
Source: Front Psychol. 2022 Sep 27;13:937366. doi: 10.3389/fpsyg.2022.937366 (PMC9552838; doi:10.3389/fpsyg.2022.937366)
Supplement: Supplementary file 1 [file Table_1.pdf]

Supplemental Material for

The cognitive process underlying ultimatum game: An eye-tracking study from a dual-system perspective

Table S1 Experimental stimuli in the experiment.

Type	Item	Responder	Proposer
inclusive payoffs	1	23	7
	2	15	15
	3	20	10
	4	16	14
	5	21	9
	6	25	5
	7	18	12
	8	17	13
	9	22	8
	10	19	11
exclusive payoffs	1	9	21
	2	4	26
	3	6	24
	4	10	20
	5	7	23
	6	8	22
	7	2	28
	8	5	25
	9	1	29
	10	3	27
